# Effect of immediate implant placement on buccal bone stability in fresh extraction sockets: A prospective study

**DOI:** 10.6026/973206300223702

**Published:** 2026-06-30

**Authors:** Fiza Khanam, Vansh Jaiswal, Keshav Tukaram Handge, Ashok Kumar Singh, Vyankatesh Sahu, Anand Rajapur

**Affiliations:** 1 Department of Oral and Maxillofacial Surgery, Jabalpur, Madhya Pradesh, India; 2 Department of Periodontology, Narsinhbhai Patel Dental College and Hospital, Visnagar, Gujarat, India; 3 Department of Dentistry, Dr Vasantrao Pawar Medical College, Hospital and Research Centre, Adgaon, Nashik, India; 4 Department of Dentistry, Viraat Ramayan Institute of Medical Sciences, Chakia, East Champaran, Bihar, India; 5 Department of Oral and Maxillofacial Surgery, Swargiya Dadasaheb kalmegh Smruti Dental College and Hospital, Hingna, Nagpur, Maharashtra, India; 6 Department of Prosthodontics, Dental College, RIMS, Imphal, Manipur, India

**Keywords:** Immediate implant placement (IIP), buccal bone stability, fresh extraction socket, alveolar bone remodeling, cone beam computed tomography (CBCT)

## Abstract

Immediate implant placement in fresh extraction sockets has been advocated to preserve buccal bone architecture and shorten overall treatment time. Therefore, it is of interest to evaluate changes in buccal bone thickness and height in 30 patients receiving immediate implants using CBCT measurements at baseline, immediately after placement and at a 6-month follow-up. Hence, a statistically significant but clinically limited reduction in buccal bone dimensions, with all implants remaining stable and no failures observed during the study period. Thus, data shows the immediate implant placement shows favorable buccal bone stability when adequate primary stability and appropriate case selection were ensured.

## Background:

Immediate implant placement (IIP) into fresh extraction sockets has gained clinical interest as it reduces treatment time and surgical interventions while preserving alveolar bone architecture [1]. Tooth extraction initiates alveolar ridge remodeling, often causing significant loss of buccal bone height and width, which affects both function and esthetics [2]. Preservation of the buccal bone plate is essential, as it is thin and more prone to resorption compared to other socket walls [3]. Immediate implant placement has been shown to better maintain alveolar ridge dimensions compared to natural healing, especially when combined with grafting techniques [4]. Clinical evidence suggests that IIP provides improved buccal bone stability compared to delayed implant placement [5]. However, IIP alone cannot completely prevent buccal bone resorption, particularly in thin biotype cases, indicating the need for adjunctive regenerative procedures [6]. Studies have reported that horizontal and vertical bone changes still occur due to natural remodeling processes even after IIP [7]. Guided bone regeneration and bone grafting at the time of implant placement help reduce buccal bone resorption and maintain ridge contour [8]. The use of graft materials such as xenografts or allografts in the implant-socket gap further supports preservation of buccal bone thickness [9]. Primary stability at the time of implant placement is a key determinant of long-term success and is influenced by socket anatomy and implant design [10]. Clinical studies have shown that IIP combined with grafting techniques in sockets with buccal defects can achieve favorable hard and soft tissue outcomes [11]. Long-term evidence indicates that IIP with augmentation can provide similar clinical success and bone stability as delayed implant placement [12]. When performed with appropriate protocols, immediate implants demonstrate high survival rates with minimal marginal bone loss [13]. Systematic reviews suggest that buccal bone preservation is comparable between immediate and delayed protocols when augmentation is used [14]. However, outcomes are influenced by patient-specific factors such as socket morphology, biotype thickness and defect severity [15]. Therefore, it is of interest to evaluate the effect of immediate implant placement on buccal bone stability in fresh extraction sockets.

## Methodology:

The collected data were entered into a spreadsheet and analyzed using a statistical software package such as IBM SPSS Statistics (IBM Corp., Armonk, NY, USA). Continuous variables were expressed as mean ± standard deviation (SD), while categorical variables were presented as frequencies and percentages. Normality of data distribution was assessed using the Shapiro-Wilk test. For normally distributed continuous variables, parametric tests were applied, whereas non-parametric tests were used for data that did not follow a normal distribution. Changes in buccal bone thickness and buccal bone height at different time intervals (baseline, immediate post-implant placement and follow-up periods) were analyzed using a paired t-test for normally distributed data or the Wilcoxon signed-rank test for non-normal data. Comparisons between independent groups, if applicable, were performed using the independent t-test or Mann-Whitney U test. Repeated measurements of buccal bone dimensions over time were evaluated using repeated-measures analysis of variance (ANOVA) or the Friedman test, followed by post-hoc analysis with Bonferroni correction where appropriate. Correlation between buccal bone stability and clinical variables such as implant diameter, primary stability and socket morphology was assessed using Pearson's correlation coefficient or Spearman's rank correlation coefficient, depending on data distribution. A multivariate regression analysis was performed to identify predictors influencing buccal bone stability following immediate implant placement. Variables with a p-value <0.20 in univariate analysis were included in the multivariate model. The level of statistical significance was set at p < 0.05 for all tests.

## Results:

A total of 30 patients (30 immediate implants) were enrolled in the study and completed the follow-up period. The mean age of the participants was 36.8 ± 8.4 years, comprising 18 males and 12 females. All implants achieved adequate primary stability at the time of placement and no implant failures were recorded during the observation period. The baseline demographic and clinical characteristics of the study population are presented in Table 1. Most implants were placed in the maxillary anterior region and all extraction sockets demonstrated intact buccal bone walls at baseline. Buccal bone thickness measurements obtained using CBCT at baseline, immediately after implant placement and at 6-month follow-up are summarized in Table 2. A gradual reduction in buccal bone thickness was observed over time. Statistical analysis showed a significant reduction between baseline and 6-month follow-up (p < 0.05). Vertical buccal bone height changes during the study period are presented in Table 3. A statistically significant reduction in buccal bone height was observed from baseline to 6 months (p < 0.05), indicating normal post- extraction remodeling despite immediate implant placement. Repeated-measures ANOVA demonstrated a statistically significant effect of time on both buccal bone thickness and height (p < 0.05). The majority of dimensional changes occurred within the first 6 months following implant placement. These findings are summarized in Table 4. Correlation analysis revealed a moderate positive correlation between primary implant stability and preservation of buccal bone thickness (r = 0.46, p = 0.01). Implant diameter showed a weak positive correlation with buccal bone stability, while implant length showed no statistically significant association. These results are shown in Table 5. Within the limitations of the study, immediate implant placement in fresh extraction sockets demonstrated favorable buccal bone stability, with limited horizontal and vertical bone loss over a 6-month period. Although physiological bone remodeling occurred, the overall preservation of buccal bone dimensions supports the clinical predictability of immediate implant placement.

## Discussion:

The present prospective study evaluated the effect of immediate implant placement on buccal bone stability in fresh extraction sockets and demonstrated limited but significant horizontal and vertical bone remodeling during the early healing period [16]. The observed reduction in buccal bone thickness over six months is consistent with previous reports indicating that physiological bone resorption occurs following tooth extraction even when an implant is placed immediately [17]. Several experimental and clinical studies have shown that immediate implant placement alone does not completely prevent buccal bone resorption, particularly in sites with thin buccal cortical plates, which supports the findings of the present study [18]. The mean horizontal buccal bone loss observed in this study was comparable to values reported in CBCT-based investigations of immediate implants placed in intact extraction sockets [19]. Vertical buccal bone height reduction noted at the six-month follow-up reflects normal post-extraction remodeling and aligns with earlier longitudinal studies assessing hard-tissue changes after immediate implant placement [20]. The majority of buccal bone dimensional changes occurred during the early healing phase, which corroborates evidence suggesting that most alveolar ridge remodeling takes place within the first three to six months following extraction [21]. The positive correlation between primary implant stability and buccal bone preservation observed in this study supports the concept that adequate primary stability plays a critical role in maintaining peri-implant bone levels [22]. Previous studies have emphasized that achieving apical and palatal anchorage during immediate implant placement contributes to improved buccal bone stability, which may explain the favorable outcomes observed in this investigation [23]. The weak but significant association between implant diameter and buccal bone stability is in agreement with reports suggesting that wider implants may provide enhanced support to the surrounding bone, although their effect remains limited [24]. Systematic reviews comparing immediate and delayed implant placement protocols have reported similar marginal bone loss outcomes when appropriate case selection and surgical techniques are employed, reinforcing the clinical validity of immediate implant placement [25]. Despite encouraging results, clinicians must recognize that buccal bone preservation is influenced by multiple factors including socket morphology, buccal plate thickness and patient-specific biological characteristics [26]. Within the limitations of the present study, immediate implant placement in fresh extraction sockets appears to be a predictable treatment modality for maintaining buccal bone stability when performed under controlled clinical conditions [27]. Recent evidence also supports these findings, where buccal bone stability following immediate implant placement showed favorable outcomes with controlled remodeling and high clinical predictability [28].

## Conclusion:

Immediate implant placement in fresh extraction sockets resulted in predictable buccal bone stability, with only limited horizontal and vertical bone remodeling during the early healing period. Adequate primary stability and proper case selection played a crucial role in maintaining buccal bone dimensions following immediate implant placement. Within the limitations of this prospective study, immediate implant placement appears to be a reliable and clinically effective approach for preserving buccal bone architecture.

## Advancement to knowledge:

This study provides clinical evidence on buccal bone dimensional changes following immediate implant placement using CBCT analysis. It highlights that immediate implant placement preserves buccal bone architecture with only limited horizontal and vertical resorption. The study reinforces the importance of primary stability as a key factor influencing buccal bone preservation. It demonstrates that most buccal bone remodeling occurs within the first 6 months after implant placement. The findings support the predictability of immediate implant placement in sockets with intact buccal bone. This study contributes to existing literature by correlating clinical parameters such as implant diameter and stability with bone preservation. It emphasizes the role of proper case selection and surgical protocol in achieving favorable outcomes. The results add to recent evidence suggesting that immediate implant placement can be a reliable alternative to delayed protocols. It also highlights the limitation that immediate placement alone does not completely prevent physiological bone remodeling. Overall, this study advances knowledge by integrating clinical and radiographic evaluation of buccal bone stability in contemporary implant dentistry.

## Figures and Tables

**Table 1:** Baseline demographic and clinical characteristics of the study population (n = 30)

**Variable**	**Value**
Number of patients	30
Number of implants	30
Mean age (years)	36.8 ± 8.4
Gender (Male/Female)	18 / 12
Implant location (Maxilla/Mandible)	21 / 9
Mean implant diameter (mm)	3.8 ± 0.4
Mean implant length (mm)	11.2± 1.3

**Table 2:** Changes in buccal bone thickness at different time intervals (mm)

**Time point**	**Mean ± SD**
Baseline	1.92 ± 0.36
Immediate post-placement	1.78 ± 0.33
6-month follow-up	1.54 ± 0.31
Mean horizontal bone loss	0.38 ± 0.14

**Table 3:** Buccal bone height changes over time (mm)

**Time point**	**Mean±SD**
Baseline	14.6 ± 1.2
Immediate post-placement	14.4 ± 1.1
6-month follow-up	13.9 ± 1.0
Mean vertical bone loss	0.7 ± 0.3

**Table 4:** Repeated-measures analysis of buccal bone dimensional changes

**Parameter**	**F value**	**p value**
Buccal bone thickness	12.4	0.001
Buccal bone height	9.7	0.004

**Table 5:** Correlation between clinical variables and buccal bone stability

**Variable**	**Correlation coefficient (r)**	**p value**
Primary stability	0.46	0.01
Implant diameter	0.29	0.04
Implant length	0.12	0.38
